# Dynamics of Quantum Correlations in Open Systems

**DOI:** 10.3390/e25020196

**Published:** 2023-01-19

**Authors:** Aurelian Isar

**Affiliations:** 1 Department of Theoretical Physics, National Institute of Physics and Nuclear Engineering, POB MG-6, 077125 Bucharest-Magurele, Romania; isar@theory.nipne.ro; 2 Faculty of Physics, University of Bucharest, POB MG-11, 077125 Bucharest-Magurele, Romania

Quantum correlations represent one of the most characteristic traits of quantum mechanics. In particular, quantum entanglement has no classical counterpart and it presents a large interest not only for testing and detecting the quantum nonlocality, but also for its crucial role played in the processing and transmission of quantum information. The unavoidable interaction of quantum systems with their environment leads to decoherence, which affects and even destroys the quantum correlations between the subsystems. Consequently, the protocols and tasks of quantum information are strongly influenced by the environment. This implies the necessity to deeply understand and characterize the dynamics of quantum correlations in open systems under the influence of quantum decoherence. The present Special Issue is devoted to addressing these important features of quantum physics, and it contains contributions studying both fundamental aspects and applications of quantum correlations in discrete- and continuous-variable quantum systems.

According to Ref. [1], the description of physical reality provided by quantum mechanics would be incomplete. A criterion for distinguishing the local and non-classical behaviours of systems is given by the Bell inequality and CHSH inequality. For three-qubit Greenberger–Horne–Zeilinger (GHZ) states, it was shown that some inequalities may violate local realism in the three-particle system, even if there are only two-particle correlations. Presently, the largely accepted opinion is that local realism has been proven wrong by experimental violations of the Bell inequality. Recently, increasing interest has been directed to the investigation of the theoretical predictions of quantum mechanics based on polarization-entangled photons. These studies show that the violation of the Bell inequality indicates the existence of entanglement in the system and that the degree of violation increases with the intensity of the entanglement in the state. In the paper “Experimental Investigation of the Robustness of a New Bell-Type Inequality of Triphoton GHZ States in Open Systems” by Jiaqiang Zhao, Meijiao Wang, Lianzhen Cao, Yang Yang, Xia Liu, Qinwei Zhang, Huaixin Lu and Kellie Ann Driscoll [2], the robustness of a new Bell-type inequality in three photon entanglement states with different levels of bit-flip noise is experimentally studied. Using spontaneous parametric down-conversion of nonlinear crystals, three-photon entangled GHZ states were prepared, and the detailed density matrices of the states were reconstructed using the over-complete quantum tomography method. They also obtained the fidelities of the entangled states. Then, a sandwich structure consisting of a half-wave plate and two quarter-wave plates was used to construct the bit flipping noise, which simulates the bit flipping noise widely existing in quantum information processing. Finally, the evolution law of state fidelity and the violation of the new Bell-type inequality against local realism with different levels of bit-flip noise were investigated. The obtained results can provide further support for evaluating the fidelity of quantum channels in quantum information processing and in security testing of ultra-dense coding.

Quantum information processing theory and experiments have been intensely developed, in particular multiphoton entanglement, which plays an important role not only in the basic tests of quantum nonlocality, but also in optical quantum computing, quantum teleportation, and quantum key distribution. A single photon is able to carry more than just a qubit of quantum information, and when two photons are entangled in more than one degree of freedom, higher-dimensional entanglement can be realized, which constitutes an attractive resource for quantum communication and quantum computation. In the paper “Preparation and Analysis of Two-Dimensional Four-Qubit Entangled States with Photon Polarization and Spatial Path” by Jiaqiang Zhao, Meijiao Wang, Bing Sun, Lianzhen Cao, Yang Yang, Xia Liu, Qinwei Zhang, Huaixin Lu and Kellie Ann Driscoll [3], the authors designed an interferometer that can couple the polarization and spatial paths of photons and prepared an entangled state of the two modes. Two-photon entangled states with high brightness and high fidelity were prepared using spontaneous parametric down-conversion technology. Next, they designed a composite interferometer and prepared two-dimensional four-qubit GHZ entanglement states for the polarization and spatial path of photons. The properties of the prepared four-qubit entangled state were analyzed by three methods: quantum state tomography, entanglement witness, and the violation of the Ardehali inequality against local realism. The experimental results proved that the prepared two-dimensional four-photon system is an entangled state with high fidelity.

The Jaynes–Cummings model was originally designed to describe the interaction of a single atom with a single-mode field. An important generalization of the Jaynes–Cummings model is the Tavis–Cummings model that describes the interaction of many two-level atoms with a single-mode of the electromagnetic field at resonance [4]. Recently, much attention has been paid to the properties of entanglement for models of light–matter interaction via models whose principal quantum system is composed of more than one two-level atom coupled with a single-mode field and also with each other via dipole–dipole and Ising-like interactions, including spin–spin interactions, trapped ions, microcavities, and dipolarly coupled molecules. In addition, the power-law potentials have provided many promising applications in theoretical and experimental physics. In the paper “Tavis-Cummings Model with Moving Atoms” by Sayed Abdel-Khalek, Kamal Berrada, Eied M. Khalil, Hichem Eleuch, Abdel-Shafy F. Obada and Esraa Reda [5], a nonlinear version of the Tavis–Cummings model is introduced for two two-level atoms interacting with a single-mode field within a cavity in the context of power-law potentials. The time evolution of entanglement of the cavity field and the two qubits through the von Neumann entropy, the entanglement between the two qubits through the concurrence, as well as the distribution of the photons through the Mandel parameter are investigated for different power-law potentials by considering the Tavis–Cummings model that describes the qubits–field interaction under the effect of velocity and acceleration.

The quantum correlations in multipartite quantum systems represent one of the most important features of quantum mechanics. Entanglement is the most studied and employed quantum correlation as a physical resource. At the same time, there exist quantum correlations in non-entangled states that can also be used in quantum protocols and tasks. The study and characterization of quantum correlations that go beyond entanglement have attracted increasing interest, and in this respect, several measures of such quantum correlations have been introduced. For continuous-variable systems, the definition of Gaussian quantum discord as two-mode Gaussian states via the mutual information and the extractable information determined by performing Gaussian positive operator value measurements has been provided. Likewise, the concept of Gaussian geometric discord for (n+m)-mode Gaussian states via Gaussian positive operator value measurements and the Hilbert–Schmidt norm was introduced. In recent years, many efforts have been devoted to finding appropriate methods to quantify these types of Gaussian quantum correlations, and various corresponding measures for them have been introduced. However, all known quantifications of these correlations for continuous-variable systems are very difficult to evaluate, mainly due to the fact that these quantifications involve measurements on one of the subsystems and an optimization process, which is difficult to perform. In the paper “A Computable Gaussian Quantum Correlation for Continuous-Variable Systems” by Liang Liu, Jinchuan Hou and Xiaofei Qi [6], a quantification *M* is proposed for bipartite Gaussian systems in terms of the covariance matrix, which avoids the measurements performed on a subsystem, as well as the optimization procedure. This Gaussian correlation measure describes the same correlation as Gaussian discord for Gaussian states but has some properties that the Gaussian discord does not possess: (1) *M* is a quantum correlation satisfying the following properties: it is 0 if and only if the state is a product state, it is locally Gaussian unitary invariant, or it is non-increasing under local Gaussian channels; (2) *M* is symmetric about subsystems; and (3) *M* can be estimated easily for any (n+m)-mode Gaussian states. As an application, the authors propose a noninvasive and repeatable quantum method for detecting intracellular temperature using the (1+1)-mode Gaussian quantum correlation *M*. The definition of *M* can be generalized naturally to any state for multipartite, multimode, continuous-variable systems, and this opens a possible way to discuss the problem of quantifying the Gaussian quantum correlations in multipartite, multimode. continuous-variable systems.

Entropy production is a basic concept in non-equilibrium classical and quantum thermodynamics. It is intimately related to the second law of thermodynamics, and therefore, it enables one to identify and quantify the irreversibility of physical processes, expressed by the generation of entropy and the dissipation of heat into the surrounding environment of the systems. However, in addition to the entropy that is flowing from the system to the environment, some additional entropy may be intrinsically generated by the process within the system, called entropy production. From the second law of thermodynamics, it follows that entropy production is always non-negative, being zero only when the system is in thermal equilibrium with its environment; consequently, it can be used as a measure of the degree of irreversibility of physical processes and to characterise a broad range of non-equilibrium phenomena. The last few years have seen a growing interest in studying the thermodynamic implications of quantum features, including the understanding of the role, properties, and evolution of entropy production in stochastic thermodynamics and in relation to the theory of open quantum systems [7,8,9]. In the case of a Markovian dynamics of open quantum systems, described by a quantum dynamical semigroup, the information is monotonically flowing from the system to the environment, and the corresponding entropy production is a non-negative quantity. In some models, a backflow of information is observed moving from the environment to the system, and this fact is usually interpreted as a signature of non-Markovianity. In the paper “Dynamics of Entropy Production Rate in Two Coupled Bosonic Modes Interacting with a Thermal Reservoir” by Tatiana Mihaescu and Aurelian Isar [10], the formalism of the theory of open systems based on completely positive quantum dynamical semigroups is employed to describe the dynamics of the rate of irreversible entropy production in a system composed of two coupled, nonresonant bosonic modes embedded in a common thermal environment. The influence of the environment is discussed in terms of the covariance matrix by taking squeezed thermal states as initial states. It is shown that the evolution of entropy production rate strongly depends on the parameters of the initial state of the system (squeezing parameter and average thermal photon numbers of the bosonic modes), frequencies of modes, parameters characterising the thermal reservoir (temperature and dissipation coefficient), and the strength of the coupling between the two modes. Using the general expression for the rate of entropy production, the behaviour of the initial state of the system, its time evolution, and the non-equilibrium stationary state of the considered system are described. Moreover, since the correlations existing in a bipartite system are determined by its entropy, and the dynamics of correlations are thus related to entropy production, a description of these two fundamental quantum characteristics is provided by comparing the behaviour of the entropy production rate and of a well-known correlation measure, namely, Rényi-2 mutual information, relative to their evolution with time and in the stationary state.

The results obtained in the papers published in the present Special Issue may provide an appreciable contribution to describing the dynamics of quantum correlations and in understanding their role in the phenomena manifested in the processing and transmission of quantum information. In addition, they present a significant potential for applications in protocols and tasks of quantum optics and quantum information.
